# 

**Published:** 1887-01-08

**Authors:** 


The Hospital.

An Institution, Family, and Parochial Journal of Hospitals, Asylums, and all Agencies for the Care of the Sick, Criticism and News.

SATURDAY, JANUARY 8TH, 1887.

### THE CHILDREN'S CORNER.

### OUR PASTIMES.

**Children's Quarterly Prize Competition.**

| | |
|---|---|
| Began .. .. .. .. .. | 1st Jan, 1887 |
| Ends .. .. .. .. .. | 26th Mar., 1887 |

PRIZES.

| | |
|---|---|
| 1st .. .. .. .. | Thirty Shillings |
| 2nd .. .. .. .. | Twenty " |
| 3rd .. .. .. .. | Ten " |
| 4th .. .. .. .. | Five " |

will be awarded to the four competitors who shall have sent in, during the thirteen weeks, the largest number of correct answers to our pastimes.

### Second Competition.

No. 8. MISSING VOWEL PUZZLE. Supply the vowels, and you will discover a well-known proverb : b r d n t h h n d s w r t h t w n t h b s h.

No. 9. CONUNDRUM. Can you tell me why a little girl's frocks, which are quite worn out and discarded, resemble children whose parents are dead ?

```
  • * •
  • * •
• • • * • • •
* * * * * * *
• • • * • • •
  • * •
  • * •
```

No. 10. ST. GEORGE'S CROSS. My lights are: a girl's Christian name; a colour; days of rest; one who performs public duties; a word that means to help; what we generally find inside a door; an animal of the deer kind. My stars read vertically and horizontally give the designation of a gentleman connected with a hospital.

No. 11. RIDDLE-ME-REE.

My *first* is a carriage of letters three,
My *second's* a vowel—an article, may be,
My *third* is a kind of covered-in cart,
My *whole* oft takes goods to the Eastern mart.

No. 12. HISTORICAL CONUNDRUM. Can you tell me which of the kings of England was by far the most unfortunate with his laundress ?

No. 13. A MEDICAL MEDLEY. Who can put the following strange medley into intelligent English : Mr.— would falsifying at the extremity of the king of Terrors, his quakers and who which what whom sent for Doctor a let (who) ter to Dr. shortened emperor behold scarlet his donkey is tance. But Doctor what Dr. pauper miss ter —would changed colour. (I will give you the clue : it begins, "Mr. Dashwood, lying at the point of death, his friends," etc.)

No. 14. A CURIOUS WORD. There is a certain English word consisting of only one syllable, but, strange to tell, if you deprive it of its first letter, it at once becomes one of two syllables. What, my little friends, is the word ?

No. 15. PUZZLE FRENCH SENTENCE. I want all my little readers to guess this ; it is so very easy :

$$\frac{\text{pir}}{\text{un}} \quad \frac{\text{vent}}{\text{vient}} \quad \frac{\text{venir}}{\text{d'un}}$$

### Results of Thirteenth Competition.

No. 68. "Prize" was found out by everyone.

No. 69. Here was another easy one, for all my little friends have discovered "Salisbury."

No. 70. This is a little problem of which most of us have heard. It looks difficult, but the answer may be easily arrived at in several ways :

Let B = length of body.

$$\text{Then tail} = 3\text{ ft.} + \frac{B}{2}$$

$$\text{and body} = 6\text{ ft.} + \frac{B}{2}$$

$$\therefore B = 12\text{ ft.}$$

$$\left.\begin{array}{l}\text{Head} = 3\text{ ft.}\\ \text{Tail} = 9\text{ ft.}\\ \text{Body} = 12\text{ ft.}\end{array}\right\} \therefore \text{Length of fish} = 24\text{ ft.}$$

No. 71. "You overcome dangers by fortitude," was guessed by everyone. My little friends appear to be now quite *au fait* at the interpretation of quaintly expressed sentences. I am not a little curious, I must confess, to see how they will get on with No. 13 above.

No. 72. Heiresses, whose wealth is protected by Chancery, resemble, of course, the rooms of a hospital, where the in-patients lie, because they are *wards!* Everyone guessed it.

No. 73. No one fell into the little trap I set. None of the birds were left on the tree, for the survivors naturally obeyed the first law of nature—self-preservation—by flying away !

No. 74. The *lights* here are

N W
U me A
R eute R
S urroun D
E xhibitor S

The initials, read downwards, give us *Nurse*, and the finals, *Wards*.

All right—Alsie, Retsibsi, Tina, T. K.
Six right—Jimmy, Florrie, Mikado.
Five right—Filomel, True Blue, Bismarck, Little Mary, Adriadne.

### Results of the First Quarterly Prize Competition.

The eventful day has at length arrived, the battle has been fought and won ! The Goddess Fortune has bestowed her favours thus :

| | Score. |
|---|---|
| Miss Burdett (Alsie), The Lodge, Porchester Square, W. .. .. .. .. .. .. .. | 51 } equal |
| Master W. M. Isbister (Retsibsi), Dunraven, Tulse Hill Park, S W. .. .. .. .. .. .. | 51 } equal |

Who will therefore divide the 1st and 2nd Prizes.

| | |
|---|---|
| Miss Ethel Maud Doggett (Tina), 62, Warwick Rd., Maida Vale, W.—3rd Prize, 10s. .. .. .. | 46 |
| Master T. Kellner (T. K.), 26, Pembroke Gardens, Kensington, W.—4th Prize, 5s. .. .. .. | 45 |

I hope those of my little contributors who have not been lucky this time, will not be disappointed. All cannot win—I only wish they could—but this being so, I hope defeat will in this instance, as it should on so many other occasions in the battle of life, be an incentive to renewed effort. Napoleon the Great used to say that the word "impossible" was not in his dictionary. I hope that all who have not this time won a prize will remember the story of the great Frenchman, and "bid defiance to defeat." I must specially compliment some of my little friends. I was sorry to see Master Tynget retire at the sixth week, for he used to answer so well. Florrie has answered well, and, like a good little girl, as I am convinced she is, has striven bravely to the end. Jimmy, too, has sent very thoughtful answers. Mikado, perhaps, takes the palm for neatness in her papers ; the way in which she writes her replies is simply excellent. Filomel, Little Mary, True Blue, Bismarck, and others, have also pleased me much. Some little people have come and gone like comets—where are Daisy and Ellie, Diosota and Ethel ? I hope to hear from them again. Ubique, Hetty, and one or two other little ones, having written me such clever little epistles, it has occurred to me that the publication of a few such nice letters would be interesting to all of us. I therefore invite each of my correspondents to enclose a little letter with their answers each week. They should not be too long, for my space is limited. They may write to me about anything they like —their studies, their little pets, their little brothers and sisters or companions, or little stories or remembrances about the hospitals. I shall award one mark for each letter I print, and these marks will be added to those given for the puzzles.

Answers, having the actual name and address (to which a pseudonym may be added for publication), must have "The Children's" Coupon attached (see advertisement pages), and be addressed to the "PUZZLE EDITOR," THE HOSPITAL, Norfolk House, Norfolk Street, Strand, W.C., not later than Thursday, 13th January, 1887. Results will appear in our issue of the 2nd January, 1887.

### "THE HOSPITAL" CHARITY BOX.

### RESULTS OF THIRTEENTH COMPETITION.

The "Dispenser" Competition has resulted as follows :—

| | Accepted Score. |
|---|---|
| Mr. L. Behenna, 50, Arundel Street, Walton, Liverpool .. .. .. .. .. .. .. | 309—1st Prize. |
| Miss E. R. Sharman, 5, Townley Park Villas, Dulwich Rise, S.E. .. .. .. .. | 284—2nd Prize. |
| Master M. J. Snell, Lynden Lodge, Elmfield Rd., Bromley .. .. .. .. .. .. | 280—3rd Prize. |

The following details show the net result of our "Charity Box" during the thirteen weeks ending to-day.

| | | |
|---|---|---|
| Prizes awarded .. .. .. .. | | £23 14 6 |
| Entrance Fees .. .. | £16 6 0 | |
| Returned Prizes .. .. | 1 1 0 | |
| | | 17 7 0 |
| Net Loss .. .. | | £6 7 6 |

The number of competitors each week was as follows :

| | | | |
|---|---|---|---|
| 1st week .. .. .. .. | 21 | | 214 |
| 2nd " .. .. .. .. | 25 | 8th week .. .. .. .. | 23 |
| 3rd " .. .. .. .. | 30 | 9th " .. .. .. .. | 21 |
| 4th " .. .. .. .. | 30 | 10th " .. .. .. .. | 13 |
| 5th " .. .. .. .. | 49 | 11th " .. .. .. .. | 25 |
| 6th " .. .. .. .. | 36 | 12th " .. .. .. .. | 15 |
| 7th " .. .. .. .. | 23 | 13th " .. .. .. .. | 15 |
| | 214 | | 326 |

This gives an average of about twenty-five per week. Whatever may be thought of this result, it must certainly be taken to prove that, to say the least, the majority of the word-puzzle competitions now so extensively advertised cannot be *bonâ fide*, because they cannot pay.

